# Targeting Cardiovascular Adverse Events of Metastatic Renal Cell Carcinoma Therapies∗

**DOI:** 10.1016/j.jaccao.2022.05.007

**Published:** 2022-06-21

**Authors:** Avirup Guha, Nicolas Sayegh, Neeraj Agarwal

**Affiliations:** a Cardio-Oncology Program, Georgia Cancer Center, Medical College of Georgia at Augusta University, Augusta, Georgia, USA; b Division of Cardiovascular Disease, Department of Medicine, Medical College of Georgia at Augusta University, Augusta, Georgia, USA; c Huntsman Cancer Institute, University of Utah, Salt Lake City, Utah, USA

**Keywords:** metastatic renal cell carcinoma, preventive cardiology, tyrosine kinase inhibitors

**Figure:**
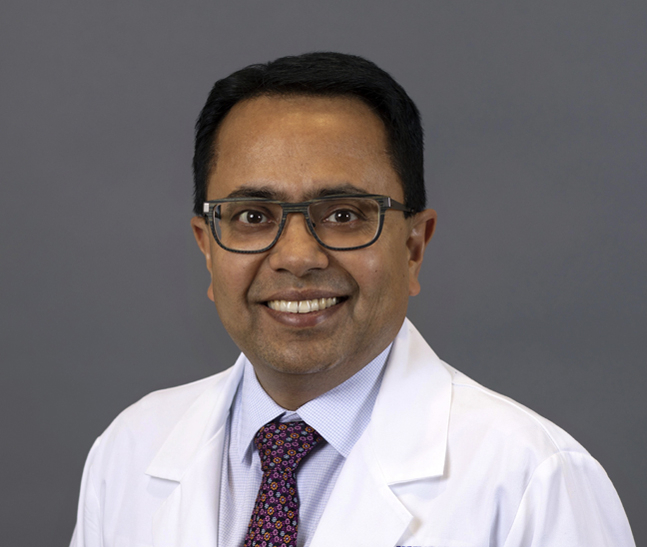

There has been significant improvement in the survivorship of patients with metastatic renal cell carcinoma (mRCC). In the cytokine era, the median overall survival was slightly longer than 1 year.^1^ Now, with contemporary treatment such as targeted therapies or immunotherapy with checkpoint inhibitors, the median survival has improved by 3- to 4-fold.^2^, ^3^, ^4^ This improvement in survival has redirected our attention from merely treating cancer at all costs to identifying the price to pay in the form of systemic toxicity. In this issue of *JACC: CardioOncology*, Chen et al^5^ present a well-executed study from Taiwan’s National Health Insurance Research Database wherein they study the major adverse cardiovascular events (MACE) associated with targeted therapy compared with cytokine therapy. The targeted drugs selected were vascular endothelial growth factor receptor (VEGFR) tyrosine kinase inhibitors (TKIs) (sunitinib, sorafenib, and pazopanib) and mechanistic target of rapamycin (mTOR) inhibitors (temsirolimus and everolimus). Cytokine therapy included interleukin-2 and interferon gamma. Overall, 81% of patients were treated with targeted therapy compared with 19% who received cytokine therapy, with the predominant type of mRCC being clear cell carcinoma (>50%). On the basis of the current guidelines for the treatment of mRCC, most patients transitioned to targeted therapy by 2012. Targeted therapy was significantly more likely to cause MACE compared with cytokine therapy (HR: 2.1; 95% CI: 1.29-3.41). This was driven predominantly by cardiovascular death, with no other specific cardiovascular events being significant. In exploratory analysis evaluating individual therapies, those receiving the mTOR inhibitor temsirolimus had a significantly increased risk for MACE compared with those receiving everolimus. Among VEGFR TKIs, sorafenib was significantly more likely associated with MACE than sunitinib or pazopanib. The factors that promoted the risk for MACE with targeted therapy were age and history of cardiovascular disease, including atrial fibrillation. This is an important, robust analysis that is well presented.

We place these findings in the context of the contemporary mRCC therapies and how this work influences the field of mRCC, as the past decade has seen a significant shift from cytokines toward VEGFR TKIs or combination regimens of VEGFR TKIs and checkpoint inhibitors (Figure 1).^6^ Prior to therapy initiation, patients are categorized by International mRCC Database Consortium (IMDC) risk groups on the basis of the following prognostic factors: <1 year between diagnosis and systemic therapy, Karnofsky performance score <80%, presence of anemia, neutrophil count >7.109/L, platelet count > 400,000/μL, and calcium level >10.2 mg/dL.^7^ Currently, there are 3 preferred first-line regimens with targeted therapies for clear cell mRCC as per the 2022 National Comprehensive Cancer Network guidelines. Axitinib, a selective VEGFR TKI, combined with pembrolizumab demonstrated superiority over sunitinib in the KEYNOTE-426 (Study to Evaluate the Efficacy and Safety of Pembrolizumab [MK-3475] in Combination With Axitinib Versus Sunitinib Monotherapy in Participants With Renal Cell Carcinoma) trial. Similarly, cabozantinib, a VEGFR TKI that also targets MET and AXL, has been approved in combination with nivolumab on the basis of data from the CheckMate 9ER (A Study of Nivolumab Combined With Cabozantinib Compared to Sunitinib in Previously Untreated Advanced or Metastatic Renal Cell Carcinoma) trial. The third combination consists of pembrolizumab plus lenvatinib, a VEGFR/fibroblast growth factor/platelet-derived growth factor receptor–α TKI, and c-KIT/RET inhibitor, following the results of the CLEAR (Lenvatinib/Everolimus or Lenvatinib/Pembrolizumab Versus Sunitinib Alone as Treatment of Advanced Renal Cell Carcinoma) trial. The efficacy of these regimens is supported by level 1 evidence in all IMDC risk groups. Notably, hypertension was the most frequent cardiovascular adverse event associated with the use of these agents across all trials, with the highest rate observed for lenvatinib plus pembrolizumab (55.4% all grades and 27.6% grade ≥3).^8^, ^9^, ^10^ It is important to note that although immune checkpoint inhibitors were not included in the study of Chen et al,^5^ their role is crucial in patients in the intermediate and poor IMDC risk groups.^4^ Preferred regimens in the 2022 National Comprehensive Cancer Network guidelines for subsequent lines include cabozantinib or nivolumab monotherapy or the combination of lenvatinib and the mTOR inhibitor everolimus.^11^, ^12^, ^13^

**Figure 1:**
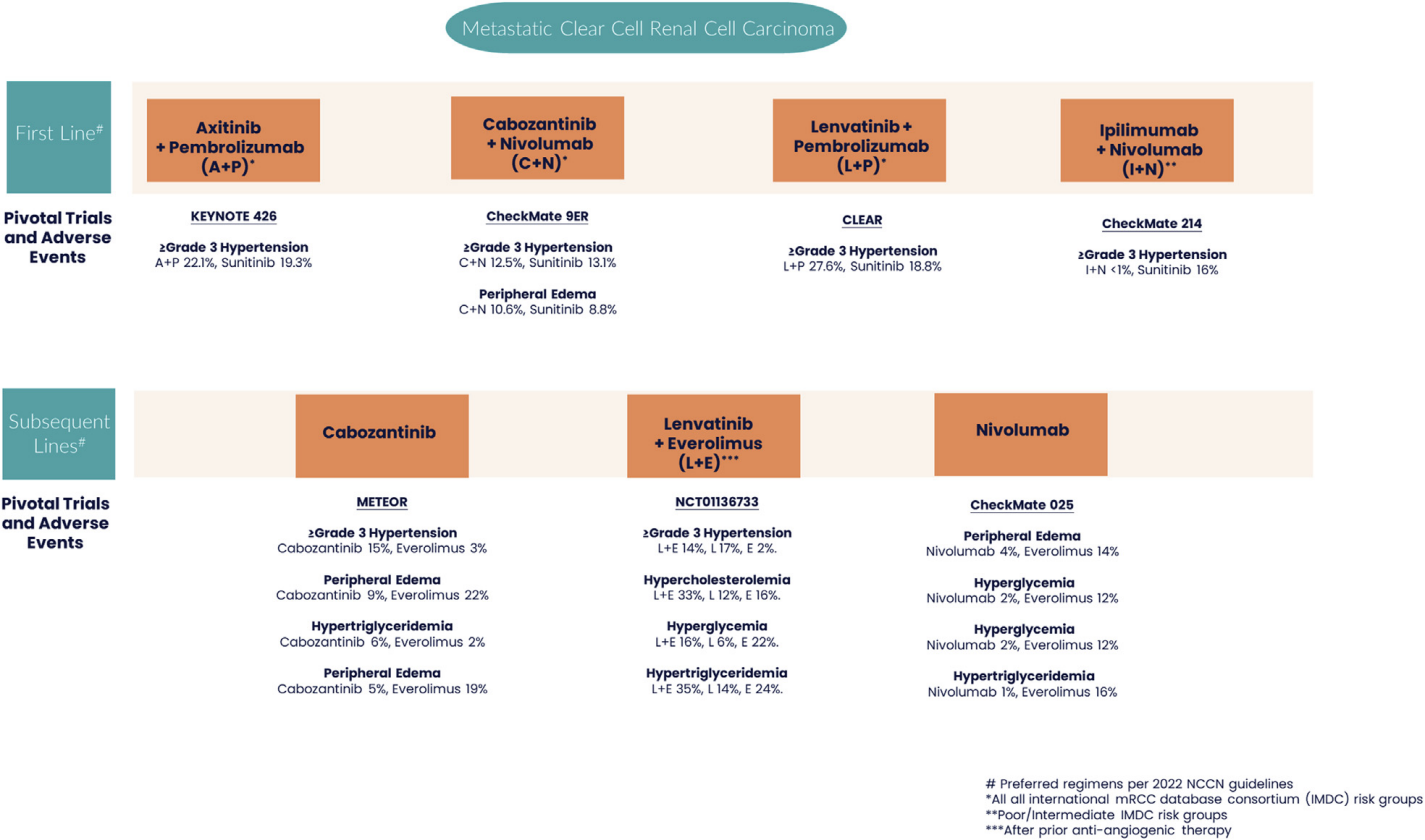
Cardiovascular Adverse Events in mRCC Treatments Cardiovascular adverse events reported in pivotal trials involving preferred treatment regimens for clear cell metastatic renal cell carcinoma (mRCC) (per the 2022 National Comprehensive Cancer Network [NCCN] guidelines) are depicted. #CheckMate 025 = Study of Nivolumab (BMS-936558) vs. Everolimus in Pre-Treated Advanced or Metastatic Clear-cell Renal Cell Carcinoma; CheckMate 214 = Nivolumab Combined With Ipilimumab Versus Sunitinib in Previously Untreated Advanced or Metastatic Renal Cell Carcinoma; CheckMate 9ER = A Study of Nivolumab Combined With Cabozantinib Compared to Sunitinib in Previously Untreated Advanced or Metastatic Renal Cell Carcinoma; CLEAR = Lenvatinib/Everolimus or Lenvatinib/Pembrolizumab Versus Sunitinib Alone as Treatment of Advanced Renal Cell Carcinoma; KEYNOTE 426 = Study to Evaluate the Efficacy and Safety of Pembrolizumab [MK-3475] in Combination With Axitinib Versus Sunitinib Monotherapy in Participants With Renal Cell Carcinoma; METEOR = A Study of Cabozantinib (XL184) vs Everolimus in Subjects With Metastatic Renal Cell Carcinoma.

Once a patient has been diagnosed and optimal guideline-directed mRCC therapy initiated, we believe that those with intermediate or favorable prognosis should be monitored for MACE during the first year, with this timing based in part on the median time to event reported by Chen et al.^5^ For those with a poor prognosis, monitoring should be decided on a case-by-case basis. In the current era of VEGFR TKI–based combinatorial treatment regimens, we believe that cardio-oncology evaluation on the basis of the ABCDE (airway, breathing, circulation, disability, and exposure) algorithm to optimize cardiovascular status prior to therapy should be strongly considered.^14^^,^^15^ Mitigating cardiovascular concerns, managing hypertension, and optimizing the management of diseases such as atrial fibrillation, heart failure, and ischemic stroke through clinical evaluation and assessment of electrocardiograms or biomarkers may minimize MACE risk more than the current standard of care.^16^ Although these approaches should be steeped in data, a priori recommendations by national and international societies could make suggestions driven by meaningful retrospective data, including this study. To make evidence-based guidelines, a prospective study is necessary, and hypotheses related to the use of a proactive approach compared with the use of a reactive approach could be tested. This could also inform subsequent clinical trials to justify more intensive cardiovascular monitoring and management. In the meantime, high-quality data as these should be a guide to insurance companies, providers, and legislators to keep patients first while approaching the problem at hand.

## Funding Support and Author Disclosures

Dr Guha is supported by the American Heart Association Strategically Focused Research Network Grant in Disparities in Cardio-Oncology (847740 and 863620). Dr Agarwal is a consultant to Astellas, AstraZeneca, Aveo, Bayer, Bristol Myers Squibb, Calithera, Clovis, Eisai, Eli Lilly, EMD Serono, Exelixis, Foundation Medicine, Genentech, Gilead, Janssen, Merck, MEI Pharma, Nektar, Novartis, Pfizer, Pharmacyclics, and Seattle Genetics; and has received institutional research funding from Astellas, AstraZeneca, Bavarian Nordic, Bayer, Bristol Myers Squibb, Calithera, Celldex, Clovis, Eisai, Eli Lilly, EMD Serono, Exelixis, Genentech, Gilead, GlaxoSmithKline, Immunomedics, Janssen, Medivation, Merck, Nektar, New Link Genetics, Novartis, Pfizer, Prometheus, Rexahn, Roche, Sanofi, Seattle Genetics, Takeda, and Tracon. Dr Sayegh has reported that he has no relationships relevant to the contents of this paper to disclose.

## References

[1] McDermott D.F., Regan M.M., Clark J.I. (2005). Randomized phase III trial of high-dose interleukin-2 versus subcutaneous interleukin-2 and interferon in patients with metastatic renal cell carcinoma. J Clin Oncol.

[2] Sternberg C.N., Davis I.D., Mardiak J. (2010). Pazopanib in locally advanced or metastatic renal cell carcinoma: results of a randomized phase III trial. J Clin Oncol.

[3] Motzer R.J., Hutson T.E., Tomczak P. (2009). Overall survival and updated results for sunitinib compared with interferon alfa in patients with metastatic renal cell carcinoma. J Clin Oncol.

[4] Motzer R.J., Tannir N.M., McDermott D.F. (2018). Nivolumab plus ipilimumab versus sunitinib in advanced renal-cell carcinoma. N Engl J Med.

[5] Chen D.-Y., Liu J.-R., Tseng C.-N. (2022). Major adverse cardiovascular events in patients with renal cell carcinoma treated with targeted therapies. J Am Coll Cardiol CardioOnc.

[6] National Comprehensive Cancer Network Kidney cancer (version 4.2022). https://www.nccn.org/professionals/physician_gls/pdf/kidney.pdf.

[7] Heng D.Y., Xie W., Regan M.M. (2009). Prognostic factors for overall survival in patients with metastatic renal cell carcinoma treated with vascular endothelial growth factor-targeted agents: results from a large, multicenter study. J Clin Oncol.

[8] Rini B.I., Plimack E.R., Stus V. (2019). Pembrolizumab plus axitinib versus sunitinib for advanced renal-cell carcinoma. N Engl J Med.

[9] Choueiri T.K., Powles T., Burotto M. (2021). Nivolumab plus cabozantinib versus sunitinib for advanced renal-cell carcinoma. N Engl J Med.

[10] Motzer R.J., Alekseev B., Rha S.Y. (2021). Lenvatinib plus pembrolizumab or everolimus for advanced renal cell carcinoma. N Engl J Med.

[11] Choueiri T.K., Escudier B., Powles T. (2015). Cabozantinib versus everolimus in advanced renal-cell carcinoma. N Engl J Med.

[12] Motzer R.J., Hutson T.E., Glen H. (2015). Lenvatinib, everolimus, and the combination in patients with metastatic renal cell carcinoma: a randomised, phase 2, open-label, multicentre trial. Lancet Oncol.

[13] Motzer R.J., Escudier B., McDermott D.F. (2015). Nivolumab versus everolimus in advanced renal-cell carcinoma. N Engl J Med.

[14] Challa A.A., Callaway A.C., Cullen J. (2021). Cardiovascular toxicities of androgen deprivation therapy. Curr Treat Options Oncol.

[15] Guha A., Gong Y., DeRemer D. (Published online October 4, 2021). Cardiometabolic consequences of targeted anticancer therapies. J Cardiovasc Pharmacol.

[16] Porta-Sanchéz A., Gilbert C., Spears D. (2017). Incidence, diagnosis, and management of QT prolongation induced by cancer therapies: a systematic review. J Am Heart Assoc.

